# Understanding and evaluating the effects of implementing an electronic paediatric prescribing system on care provision and hospital work in paediatric hospital ward settings: a qualitatively driven mixed-method study protocol

**DOI:** 10.1136/bmjopen-2015-010444

**Published:** 2016-02-03

**Authors:** Albert Farre, Carole Cummins

**Affiliations:** 1 Institute of Applied Health Research, University of Birmingham, Birmingham, UK; 2 Research and Development, Birmingham Children's Hospital NHS Foundation Trust, Birmingham, UK

**Keywords:** Electronic Prescribing, Computerized Physician Order Entry System, PAEDIATRICS, Ethnography, Qualitatively-driven mixed-method research

## Abstract

**Introduction:**

Electronic prescribing systems can improve the quality and safety of healthcare services, but their implementation is not straightforward and may create unexpected change. However, the added complexity of paediatric prescribing (eg, dose calculations, dilutions, manipulations) may pose additional challenges. This study will aim to (1) understand the complex organisational reality of a paediatric hospital in which a new electronic paediatric prescribing (ePP) system will be introduced; (2) describe ePP-related change, over time, in paediatric hospital ward settings; (3) explore staff perspectives in relation to currently established practices and processes; and (4) assess the impact of ePP on care provision and hospital work from the perspective of paediatricians, paediatric nurses and managers.

**Methods and analysis:**

A qualitatively driven mixed-method approach will be adopted, including 3 inter-related substudies. The core component of the study will be qualitative (substudy 1): we will use ethnographic research methods, including non-participant observation in wards and informal conversational interviews with members of staff. In addition, the design will include 2 embedded supplementary components: a qualitative 1 (substudy 2) based on in-depth interviews and/or focus groups with paediatricians, paediatric nurses, paediatric pharmacists/pharmacy technicians and managers; and a quantitative 1 (substudy 3) in which a staff survey will be developed and administered before and after the ePP implementation. Analytic themes will be identified from ethnographic field notes and interview data. Survey data will be analysed using descriptive statistics and baseline and follow-up data compared to establish impact evaluation measures.

**Ethics and dissemination:**

A favourable ethical opinion has been obtained from a National Health Service (NHS) Research Ethics Committee (15/SS/0157). NHS research governance approval has been obtained at the relevant hospital site. The results of the study will be disseminated through conferences and peer-reviewed journals, as well as fed back to those involved in clinical practice and policy development at the study site.

**Strengths and limitations of this study**

- We will explore the impact of a new electronic paediatric prescribing system before, during and after its implementation. We are not aware of similar longitudinal ethnographic studies.
- By describing and understanding how the implementation process unfolds over time, we also expect to provide explanations relevant to quality and safety outcomes.
- We will develop and test new measures of impact drawing on health professionals’ and managers’ perspectives.
- Key quantitative quality and safety outcomes demonstrated in other studies and settings (such as impact on medication error rates) will not be measured as part of this study. However, other studies in the same setting will cover these aspects.
- The complexity specific to paediatric prescribing and paediatric organisations might limit the transferability of some of our findings.

## Introduction

It has been widely acknowledged that the introduction of new information and communication technology in healthcare—or Healthcare Information Technology (HIT)—which usually implies full replacement of a complex set of well-established practices, objects and interactions that have evolved over the years—is not always free from critical and unintended consequences.^1^

Despite the fact that the incorporation of new HIT is often seen as a simple, safe, cheap and effective solution, practical experience of HIT implementation shows how the interplay between new HIT and existing configurations and practices of healthcare work is complex and may involve potentially disruptive effects or result in emergent outcomes.^2^ Likewise, there is increased recognition of the potential for technological interventions to be compromised or fail if they do not integrate with existing work.^3^ ^4^ Examples of this can be found in various areas such as telemedicine and e-health,^5^ telephone triage expert systems,^6^ computer decision support systems in emergency and urgent care,^7^ computerised systems for the production of plans of care for hospital inpatients^8^ or electronic medical record technologies.^9^

As a new HIT, electronic prescribing (ePrescribing) or Computerized Physician Order Entry Systems (CPOE) has been identified as a means to improve quality and safety in healthcare services delivery.^10^ ^11^ However, it has also been acknowledged that the implementation of such systems is not straightforward and may create unexpected change—good or bad—once in use.^12^ Some of these unintended and undesired consequences are social in nature, with various potentially adverse effects on clinical workflows^13^ and collaborative working^11^ such as increased data entry tasks and redistribution of work and time for patient care^14^ or reduced interactions between nurses and doctors.^15^ As work practices change hospital staff have to learn new ways of working, which can also result in resistance to use HIT or the development of ‘workarounds’ where technology is used in ways other than intended.^7^ ^16^ ^17^ Examples of these can also be found in recent contributions to the field.^11^ ^18–20^

In paediatrics, the need for calculations, dilutions and manipulations of paediatric medicines, together with a need to dose on an individual patient basis using age, gestational age, weight and surface area, means that they are more prone to medication errors at each stage of the medicines management process.^21^ In recent years, a growing body of evidence has contributed to the emerging field of electronic paediatric prescribing (ePP).^22–26^ Studies to date have largely focused on medication errors,^27^ ^28^ evaluation and response to medication dosing alerts^29^ ^30^ and on evaluating the potential of ePP to reduce dosing and other medication errors.^31^ ^32^ However, this does not preclude any organisation that intends to implement ePP from facing similar challenges to the ones already described in the wider arena of electronic prescribing or the even wider field of HIT implementation. Therefore, there is still the need to identify and address such challenges in the particular case of ePP.

Inductive exploratory approaches of a qualitative nature can make an important contribution to support the implementation and development of ePrescribing systems in specific contexts^14^ ^33^ ^34^ and have significantly contributed to inform initiatives such as the ePrescribing Toolkit for National Health Service (NHS) Hospitals^35^ developed by the National Institute for Health Research (NIHR)-funded ePrescribing Research Programme.

In particular, ethnographic studies have often identified ‘hidden’ or less obvious aspects of ePrescribing impacting on key issues such as quality and safety. For instance, Swinglehurst *et al*^36^ identified the contribution of receptionists and administrative staff to quality and safety in repeat prescribing in the context of general practices; Bossen and Markussen^37^ identified the capacity of electronic prescribing devices to order information, stipulate action and coordinate interaction; and recently, Dixon-Woods *et al*^38^ identified the risk of focusing attention on aspects of patient safety made visible by electronic prescribing systems at the expense of other, less measurable but nonetheless important, concerns.

An ethnographic approach to ePP implementation is particularly well suited to explore the implications of ePP on health professionals’ work practices. Informal work practices are expected to vary, but assessing and tracing changes and effects over time is likely to pay significant returns for the implementing organisation, users and the quality of patient care.^16^ In addition, ethnographic data may also yield valuable insights into the organisational contextual factors^39^ of ePP and the understanding of why errors happen and how new ePP systems support the practices and processes underlying such errors.^40^

Beyond that, as highlighted by Barber,^12^ a mixed-method evaluation of electronic prescribing systems allows the measurement of effects, together with an understanding of why these effects are as they are, and issues which need to be addressed to develop the system.

In keeping with the latter, and taking into account recent findings and lessons learned from national and international research on ePrescribing suggesting the importance of process evaluations and the key role of social and organisational factors, alongside the technical characteristics of systems, in facilitating successful implementation of ePrescribing and other HIT,^11^ ^41^ we propose a qualitatively driven mixed-method process and impact evaluation of the implementation of a new ePP system in a paediatric hospital.

In particular, we will explore how this technology is enrolled in everyday working practices in the ward setting and how such practices change over time through ethnographic research, complemented at various stages by focus groups, interviews and questionnaires to enable a more encompassing identification and understanding of the social processes through which ePP is brought into action. By understanding how everyday working practices change, we expect to be able to identify if, why and how the introduction of a new ePP system impacts on healthcare delivery in the ward setting.

This study will be the first ethnographic research to date known to the authors on the implementation of ePrescribing to explore the phenomenon before and over time throughout the implementation process, and the first to be undertaken in a paediatric hospital in the UK.

We expect that such approach will enable us to identify and examine a range of implications that still remain unaddressed in paediatrics and which cannot be elicited from sources such as audit data, routine metrics and statistics or incident-reporting systems.^42–44^ The study will engage with the production of ‘soft intelligence’^44^ in that we will aim to address complex data about quality and safety that cannot be straightforwardly classified and/or quantified, particularly those that lie between acts of recording/reporting and actual practices.^38^ ^42^ ^45^ ^46^ In doing so, we will also seek to consider whether or not key issues raised by previous ethnographic research^36–38^ and other qualitative studies in the field of ePrescribing 14 33–35 may appear equally relevant, nuanced or absent in the context of a paediatric organisation.

In addition, this study will contribute to a more encompassing programme of work, which aims to assess the realisation of the expected benefits and outcomes of ePP by integrating findings from this and two other studies (one looking at key safety outcomes using routine statistics, and another focusing on efficiency) that will be undertaken alongside, but independently of, this study at the same hospital site over approximately the same period of time. In this context, we expect our findings to provide explanations relevant to key outcomes measured by these studies, as well as to contribute new measures of impact drawing on health professionals’ and managers’ perspectives.

### Aim and objectives

The overall aim of this study is to help improve the quality and safety of healthcare delivery in paediatric hospital ward settings by generating evidence to facilitate the implementation of ePP in such settings.

Our objectives are:

1. To understand the complex organisational reality of a paediatric hospital in which a new ePP will be introduced.
2. To describe the nature of ePP-related change processes by examining how practices and procedures change, or are replaced, over time in paediatric hospital ward settings as the system is implemented.
3. To explore staff perspectives (including paediatricians, paediatric nurses, paediatric pharmacists/pharmacy technicians and managers) in relation to currently established practices and processes involved in the prescribing and administration of medicines in a paediatric hospital ward setting.
4. To assess the impact of implementing ePP on care provision and hospital work in a paediatric hospital ward setting from the perspective of paediatricians, paediatric nurses and managers.

## Methods and analysis

### Design and data collection

**Figure 1:**
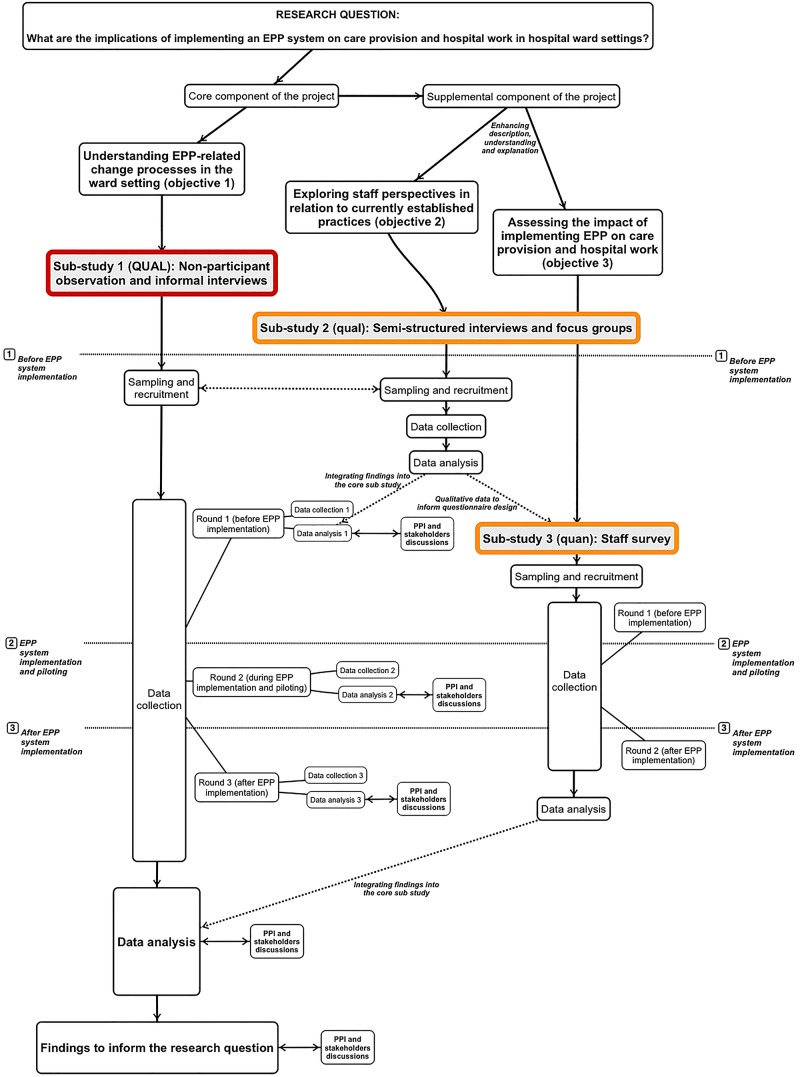
Visual model of the design. EPP, electronic paediatric prescribing.

To meet the aforementioned aims and objectives, we will adopt a qualitatively driven mixed-method approach^47^ ^48^ involving three inter-related substudies (figure 1).

A mixed-method design, in contrast to multimethod research, consist of one project, known as the core project, which is a complete method in itself, and a supplemental strategy (or more than one) consisting of a different type of data or analysis, that is not comprehensible or interpretable apart from the core project.^47^

For our project, we will use an embedded design^49^ including a primary qualitative study and two embedded supplementary substudies, qualitative and quantitative, respectively (see figure 1).

Therefore, the rationale that will guide the conduct of the study will draw on qualitative reasoning and the overall theoretical drive will remain inductive.^48^ In this context, quantification and statistical analysis will play a subordinate role to the overall interpretative framework and thus contribute to the production of descriptions, explanations and emerging theories.

#### Substudy 1 (primary qualitative component): understanding change in the ward setting

As the primary component of our design, this qualitative study will provide the overall rationale for the interpretation of findings and will mainly contribute to meeting objectives 1 and 2 by *identifying, describing and understanding how professionals’ interactions and practices are transformed, or replaced, over time as ePP is implemented*.

We will focus on how health professionals make the prescribing and administration of medicines practically workable in a changing organisational context and what factors enable or inhibit the successful introduction of ePP in a paediatric ward setting.

To do this, we will use ethnographic research methods, including non-participant observation,^50^ informal conversational interviews^51^ and relevant documentary data (eg, relevant ward documentation, hospital policies, reports and guidance) will be collected where appropriate. The foundations of our proposal engage with grounded theory and ethnomethodology approaches to ethnographic work.

Fieldwork will take place in three phases:

1. *Before ePP implementation*: The first phase will take place prior to the ePP implementation, involving approximately 150 h of observation over the past 4–5 months before implementation/piloting of the system.
  - Primary focus: describing and understanding the current set of practices and organisational reality surrounding the prescribing and administration of medicines, as well as exploring the social organisation of bringing paper-based prescribing into action.
  - Data collection priorities:
    - Formal interviews will be conducted with key informants, including ePP project leads in the hospital for medical, nursing, pharmacy and IT areas, as well as any relevant managers.
    - We will shadow paediatricians (as they prescribe medicines on the wards), paediatric nurses (as they go about prescription checking, preparation and administration of medicines) and paediatric pharmacists/pharmacy technicians (as they undertake stock checks and drug charts checks on the wards) across a general paediatric, a general surgical and a highly specialised ward.
    - We will follow the course and use of paper drug charts through the prescribing, preparation and administration of medicines in (or beyond) the ward settings.
    - We will undertake textual analysis of key documentary data, such as relevant hospital policies and guidance documents.
2. *During ePP implementation/piloting*: The second phase will take place during the implementation and/or piloting of the new ePP system, involving approximately 100 h of observation.
  - Primary focus: exploring how everyday working practices are transformed or replaced as ePP is being implemented, as well as identifying emerging problems and solutions.
  - Data collection priorities:
    - We will observe regular meetings of the ePP implementation group (including members from medical, nursing, pharmacy and IT areas) as the system is piloted and rolled out.
    - We will shadow paediatric prescribers, paediatric nurses, and paediatric pharmacists and pharmacy technicians as they interact with the new ePP system and follow the course of these practices through the prescribing, preparation and administration of medicines in the pilot ward.
    - Observations will include at least one non-pilot ward to allow for comparisons and identify any emerging aspects that can facilitate/hinder the implementation.
3. *After ePP implementation*: The last phase will take place after ePP is implemented in all wards, involving approximately 100 h of observation over the following 6 months after the system is rolled out in all wards.
  - Primary focus: establishing how well the new ePP system is integrated into (and sustained through) everyday working practices.
  - Initial data collection priorities:
    - We will observe follow-up meetings of the ePP implementation group (including members from medical, nursing, pharmacy and IT areas).
    - We will undertake textual analysis of changing hospital policies and/or guidance documents.
    - We will shadow paediatric prescribers (including medical and non-medical prescribers, as they prescribe medicines on the wards or remotely), paediatric nurses (as they go about prescription checking, preparation and administration of medicines) and paediatric pharmacists/pharmacy technicians (as they undertake stock checks and drug charts checks on the wards or remotely) and observe how the new ePP system is used in practice across the observed wards.

Briefing and debriefing sessions with the research team will be held before and after each data collection session. Within each phase, data collection sessions will take place in two separate rounds. After each round, the researcher will engage in periods of more focused data analysis and define the priorities for the next round of data collection.

Alongside these phases, we will undertake a systematic review of qualitative studies, which will further inform the conduct of the study. However, a separate protocol will be produced for this specific piece of work and will be registered as appropriate.

#### Substudy 2 (supplementary qualitative component): exploring staff perspectives on currently established practices

This substudy will mainly contribute to meeting objective 3, but also inform objectives 1 and 2, by eliciting and analysing staff (including paediatric prescribers, paediatric nurses, paediatric pharmacists/pharmacy technicians and managers) views and experiences in relation to currently established practices and processes involved in the prescribing and administration of medicines in the ward, as well as their expectations, fears and basic assumptions in relation to the new ePP system.

To do this, we will use qualitative methods, including individual in-depth interviews^52^ and, where possible, focus groups.^53^ ^54^

#### Substudy 3 (supplementary quantitative component): measuring impact of ePP on patient care and hospital work in the ward setting

This substudy will mainly contribute to meeting objective 4 by measuring the impact of ePP on hospital work and patient care from the perspective of the staff involved in care provision in the ward setting.

To do this, we will use a before-and-after design to administer an online self-completion questionnaire, aimed at measuring staff perceptions in relation to each of the items included, to staff involved in the ward setting.

The questionnaire will be developed based on: (1) qualitative data emerging from substudy 2, which will allow us to devise an instrument that measures meaningful domains for the target population in an accessible and inclusive way; (2) the hospital's own working framework of expected benefits/outcomes; and (3) NHS England guidance on the benefits of ePrescribing.^55^

As a result, we expect the resulting set of items to include a combination of:

- Expected practice-related outcomes (eg, Do paediatricians and paediatric nurses feel their practice will be/is safer?).
- Expected changes in working practices and procedures (eg, Do paediatricians feel that remote access will ease/has eased their workload?).
- Unexpected emerging issues (eg, Do paediatric nurses feel that the technology will facilitate/facilitates their interaction with pharmacists and doctors to check/amend prescriptions in a more timely fashion?).

In addition, the resulting set of self-developed items will be complemented by items from a measurement instrument based on Normalization Process Theory (NoMAD)^56^ in order to also measure other generic aspects that can be associated with the implementation process.

Experts in the field will be asked to check the face and content validity of the questionnaire. The reliability of the questionnaire will be assessed through individual interviews with 10 respondents after completion, in which the survey questions will be repeated and the variance between written responses and the interview data will be calculated. Difficult or confusing questions will be identified and discussed, as well as the clarity and wording of the instructions. The questionnaire will be amended where appropriate and then administered to the rest of the sample, before and after the implementation of the new ePP system.

### Participants and sampling

The overall sampling strategy will require the following combination of non-random and random strategies:

In substudy 1, we will focus on those members of staff directly or indirectly involved in the prescribing and administration of medicines, in different shifts, in selected wards. We will use a theoretical sampling strategy^57^ minimising the differences between cases in the first instance, and then subsequently maximising the differences between cases where possible, although intensive care units and oncology wards will be excluded from this study, due to their distinct dynamics and to avoid overlaps with ongoing research specifically focusing on such settings.

In substudy 2, we will focus on three groups of staff directly involved in the prescribing and administration of medicines in the ward (ie, doctors, nurses and pharmacists) in addition to any relevant managerial roles. We will use a purposive maximum variation sampling strategy^51^ which involves sampling a wide range of cases to get variation on dimensions of interest, which in this case are types of wards and roles involved in the prescribing and administration of medicines on the ward.

In substudy 3, we will focus on staff primarily involved in direct patient care on the ward (ie, nurses, doctors and other relevant health professionals) and staff primarily involved in the organisation and functioning of the ward (ie, ward managers and clerical staff). We will use a stratified sampling strategy, which will allow us to define groups that are homogeneous with respect to one or more characteristics and then select a random sample within each group.

As both substudies 1 and 2 will rely on purposive sampling, the sample size should ideally be determined by saturation, which is considered the gold standard by which purposive sample sizes are determined.^58^ However, as an estimate for illustrative purposes, we can anticipate a minimum sample of 2 wards and 30 informal interviews for substudy 1, and 6–12 interviews and 3–6 focus groups for substudy 2. As for substudy 3, although sample size will be appropriately determined at the early stages of the questionnaire design, following the recommendations formulated by Borg and Gallo (1989, cited in ref. 59), we estimate a sample size of about 100 participants for the major subgrouping of the survey (ie, staff primarily involved in direct patient care) and 20–50 for minor subgroupings (ie, staff primarily involved in the organisation and management of the ward).

The overall inclusion/exclusion criteria are the following:

- Inclusion criteria: members of staff; over the age of 18; able to give consent.
- Exclusion criteria: refusal of consent; unable to give consent; withdrawal of consent.

### Data analysis

Qualitative and quantitative data will be connected and integrated at various stages. However, as we are using a qualitatively driven design, the rationale for the overall data analysis process and the interpretation of findings will draw on qualitative reasoning.

Ethnographic field notes will be anonymised and analytic themes will be identified, categorised and refined following the principles of the constant comparative method^60^ and other procedures from first-generation grounded theory, such as coding and memoing.^60^ Data collection and data analysis will take place concurrently, so that issues raised in earlier rounds of fieldwork can be explored in subsequent ones, and analytic categories subsequently refined and further developed. Documentary data collected during ethnographic fieldwork will be analysed using techniques from textual analysis.^61^ The coding and analysis process will be aided by QSR NVivo V.10 software.

The overall data analysis will also be theoretically informed by Normalization Process Theory (NPT)^4^ ^3^ ^62^ which is a sociological theory concerned with the social organisation of the *work* (implementation), of making practices routine elements of everyday life (embedding), and of sustaining embedded practices in their social contexts (integration).^62^ The emerging coding framework will be put into dialogue with the constructs of NPT in order to enhance and expand the interpretative framework in the light of an explanatory theory that enables structured comparative inquiry and that therefore may facilitate the transferability of our findings to other settings and cases.

Audio-recorded data from in-depth interviews and focus groups will be transcribed verbatim, anonymised and then subjected to thematic analysis.^63^ ^64^ These findings will inform the design of the questionnaire and then will be integrated into the overall qualitative data set.

Quantitative data from the baseline survey will be processed, analysed using descriptive statistics and the results used to expand or complement the interpretation of qualitative data in subsequent rounds of data analysis.^65^ Quantitative data from the follow-up survey will be processed, integrated into the quantitative data set and analysed. Baseline measures will then be compared with follow-up measures to establish the resulting impact evaluation measures. All statistical analyses will be performed with IBM SPSS Statistics V.21 software.

## Ethics and dissemination

### Ethical considerations

The study received a favourable ethical opinion from an NHS Research Ethics Committee (15/SS/0157), and NHS research governance approval was obtained at the relevant hospital site.

Patient and staff information sheets will be distributed on the wards and clinical areas before and during the observations. Before the observations, the researchers will spend time on each selected ward across all shifts and aim to be able to speak to the majority of staff to make them aware of the observations, respond to any queries about the study and hand them information sheets. In addition, information sheets will be sent through the Trust email, and posters announcing the observations will be put on the selected wards before and during the observation sessions. Participation in the study will be voluntary, and participants will be able to decline or withdraw at any time. Where possible, the consent will be written; however, there might be unexpected people at the ward at the time of the observations from whom it may not be possible to obtain consent in advance. In such instances, we will seek verbal consent.

Although patient information will not be recorded/considered for the study and parents/children are not the subject of our observations, they both may be present in a range of situations. In these instances, we will prompt the relevant clinician to introduce the researcher, who will then seek their verbal consent.

For the interviews, potential participants will be directly approached by the researcher, who will hand them the relevant information sheet. Written consent will be sought after leaving at least 24 h to consider their participation.

Finally, the questionnaire will be administered electronically and members of staff will initially be invited to participate via email. An information sheet will be included in the initial correspondence, and the consent form will be available as the first page of the online survey. The contact details of the research team will be provided with the initial correspondence and members of staff will be free to contact a named researcher to clarify the information given or request further information.

### Dissemination of findings

The study results will be disseminated through conferences and peer-reviewed journals, as well as fed back to those involved in clinical practice and policy development at the study site, so that they can be better integrated into routine clinical practice.

To this end, the study will incorporate a participatory component which will combine engagement with (1) key stakeholders at the hospital, and interested members from (2) the Collaboration for Leadership in Applied Health Research and Care (CLAHRC) West Midlands patient and public involvement (PPI) advisory group; (3) the hospital's young people advisory group; and (4) the hospital's special interest group on ePrescribing. This process will be operationalised through various discussion sessions, the frequency and dynamics of which will be determined with the participants. We will aim to discuss the findings before the end of each data analysis phase of substudy 1 (ie, before, during and after implementation) as well as during and after the overall data analysis phase (see figure 1). The purpose of these sessions is twofold: on the one hand, we intend to use the views of those involved to enhance and challenge our coding framework and interpretation of the data. On the other hand, we expect that the participation process will increase the meaningfulness and encourage ownership of the findings for those with responsibility for implementation decisions as well as those affected by the intervention.
